# Supplementary material for: Large-scale analysis of whole genome sequencing data from formalin-fixed paraffin-embedded cancer specimens demonstrates preservation of clinical utility
Source: Nat Commun. 2024 Sep 4;15:7731. doi: 10.1038/s41467-024-51577-2 (PMC11374794; doi:10.1038/s41467-024-51577-2)
Supplement: Supplementary file 1 — Supplementary Information [file 41467_2024_51577_MOESM1_ESM.pdf]

Supplementary Information

Large-Scale Analysis of Whole Genome Sequencing Data from Formalin-Fixed Paraffin-Embedded Cancer Specimens Demonstrates Preservation of Clinical Utility

Table of Contents

Section S1. Supplementary Methods **4**

Section S1.1. Sample collection and processing **4**

Genomics England Cohort 4

Oxford Cohort 4

PARTNER/PBCP Cohort 4

Section S1.2. Nucleic acid extraction **4**

Genomics England Cohort and PARTNER/PBCP Cohort 4

Oxford Cohort 5

Section S1.3. Library preparation **5**

Genomics England 5

Oxford Cohort 6

PARTNER/PBCP Cohort 6

Section S1.4. Whole genome sequencing and alignment **6**

Section S1.5. Variant annotation **6**

Genomics England 6

Oxford and PARTNER/PBCP Cohorts 6

Section S1.6. Quality Assessment **7**

Section S1.7. Variant allele frequency and mutational burden **7**

Section S1.8. FFPEimpact formula **7**

Section S1.9. Using FFPE indel artefact to salvage HRDetect **8**

Section S1.10. Calling of somatic copy number drivers **8**

Tables ***9***

Supplementary Table 1: Overview of samples used for analysis – Genomics England Cohort **9**

Supplementary Table 2: Overview of samples used for analysis – Oxford Cohort **9**

Supplementary Table 3: Overview of samples used for analysis – PARTNER/PBCP Cohort **9**

Supplementary Table 4: Average sequencing coverage **9**

Supplementary Table 5: Average coverage and alignment metrics (by sample preparation) – Genomics England Cohort **9**

Supplementary Table 6: Correlation of cancer cell content to VAF **10**

Supplementary Table 7: Copy number driver event comparison between FF and FFPE (amplifications) **10**

Supplementary Table 8: Copy number driver event comparison between FF and FFPE (homozygous deletions) **10**

Supplementary Table 9: Summary of SBS57 presence in FF samples **11**

Supplementary Table 10: Mutation burden comparison in Oxford and PARTNER/PBCP Cohorts **11**

Supplementary Table 11: Driver Gene Mutations in Oxford Cohort **11**

Supplementary Table 12: Matched Actionable Mutations in Oxford Cohort **12**

Supplementary Table 13: Matched Domain 1 Mutations in PARTNER/PBCP Cohort **13**

Supplementary Table 14: Overview comparison between FF and FFPE whole genome sequencing 13

Figures 14

Supplementary Figure 1: Tumour purity between different sample preparation methods 14

Supplementary Figure 2: Proportional contribution of each organ type to total samples 14

Supplementary Figure 3: Somatic variants in potentially actionable genes 15

Supplementary Figure 4: Relationship between artefactual signatures and FFPEimpact score 16

Supplementary Figure 5: Association between DNA extraction protocol and FFPEimpact score 17

Supplementary Figure 6: Mutational burden analysis for Oxford cohort 17

Supplementary Figure 7: Mutational burden analysis for PARTNER/PBCP cohort 18

Supplementary Figure 8: TruSeq FFPE research workflow 18

Full Collaborator Project Acknowledgements 19

Personalised Breast Cancer Program (PBCP) 19

Project team 19
Past members 19
Site Principal Investigators and team 19
Acknowledgements 19
Funders 19

PARTNER Trial 20

Project team 20
Trial Management Group 20
Site Principal Investigators 20
Acknowledgements 20
Funders 21

References 22

Section S1. Supplementary Methods

Section S1.1. Sample collection and processing

Genomics England Cohort

Sample data was obtained from Genomics England Limited (GEL) through the 100,000 Genomes Project (100KGP) cancer pipeline (version 8). Tumour samples were collected from patients undergoing biopsy or surgical resection and processed by National Health Service (NHS) Genomic Medicine Centres according to GEL guidelines. Matched normal samples were obtained from peripheral blood. Solid tumour samples were fixed and stored as fresh frozen (FF) or formalin-fixed paraffin-embedded (FFPE). All FFPE samples were fixed using 10% Neutral Buffered Formalin (NBF). Sample processing schedules included urgent, overnight and extended (>48 hours).

Oxford Cohort

Tumour samples were collected from patients undergoing surgical resection with curative intent at the Oxford University Hospitals Foundation Trust. Samples were prepared as both FF and FFPE samples as per the usual protocol in NHS diagnostic laboratories depending on the tissue type. FF specimen were prepared from at least one 5mm punch, with the FFPE block being taken from the area surrounding the FFPE punch. FFPE samples underwent fixation in 10% NBF with sodium chloride or phosphate for routine sectioning. The median time between specimen collection and DNA extraction was 56.2 days for FFPE specimen.

PARTNER/PBCP Cohort

FFPE samples were collected from patients consented to the PARTNER neoadjuvant clinical trial for triple negative breast cancer patients. FFPE samples underwent fixation in 10% NBF for routine sectioning.

FF samples for tumour samples were collected from 14 patients co-consented into the Personalised Breast Cancer Program (PBCP) at Cancer Research UK Cambridge Institute/Cambridge University Hospitals Foundation Trust. FF samples were prepared as per the study protocol.

Section S1.2. Nucleic acid extraction

Genomics England Cohort and PARTNER/PBCP Cohort

Sample handling and DNA extraction was performed as outlined in the Genomics England Sample Handling Guidance Documentation. DNA was extracted from FFPE samples using QIAamp® DNA FFPE Tissue Kit (QIAGEN, Hilden, Germany) or truXTRAC® FFPE DNA Kit (Covaris, Woburn, MA, USA), following manufacturer instruction. DNA was extracted from FF samples according to individual laboratory standard procedure. Illumina TruSeq PCR-Free and Nano library preparation kits (Illumina, San Diego, CA) were used for FF samples with sufficient (>2µg) and insufficient (500ng-2µg) DNA, respectively.

Oxford Cohort

For peripheral blood, genomic DNA samples were extracted with QIAamp DNA Mini Kit (Qiagen, Hilden, Germany) as per the manufacturer's instructions. For FF tissues, genomic DNA was extracted from approximately 30mg using the All Prep DNA/RNA Mini Kit (Qiagen, Hilden, Germany) following the manufacturer's protocol. For FFPE tissues, two 1 mm diameter core punches were collected from the paraffin block and DNA was extracted by the truXTRAC FFPE DNA Kit (Covaris, Woburn, MA) according to the manufacturer's instructions for extraction using a M220 ultrasonicator (Covaris), performing an overnight incubation with proteinase K at 56°C.

DNA and RNA were extracted from FFPE samples using the FormaPure XL Total (Beckman Coulter Life Sciences) on the BioMek FXP platform (Beckman Coulter, Life Sciences). DNA/RNA from Fresh frozen tumour samples were extracted manually using the AllPrep DNA/RNA Mini Kit (Qiagen: Cat No: 80204). Extracted DNA/RNA were quantitated by Qubit broad range kits (Thermofisher Scientific).

Section S1.3. Library preparation

Genomics England

Illumina TruSeq PCR-Free and Nano library preparation kits (Illumina, San Diego, CA) were used for FF samples with sufficient (>2µg) and insufficient (500ng-2µg) DNA, respectively. FFPE libraries were prepared with the a modified version of the TruSeq Nano kit. Two DNA repair steps were included after DNA fragmentation. The first repair step contains Uracil DNA Glycosylase and Endonuclease IV to remove deaminated cytosine residues and to hydrolyse the backbone at the abasic site. The second repair step contains RecJ to remove single stranded DNA. DNA repair steps were followed by end repair, size selection, adapter ligation and eight PCR cycles were performed on library samples. See Supplementary Figure 8 for the TruSeq FFPE research workflow.

Oxford Cohort
Samples were processed as per samples in Genomics England cohort. Illumina TruSeq PCR-Free preparation kit (Illumina, San Diego, CA) was used for FF samples and FFPE libraries were prepared with the a modified version of the TruSeq Nano kit.

PARTNER/PBCP Cohort
Samples were processed as per samples in Genomics England cohort without initial QC cut offs. Illumina TruSeq PCR-Free preparation kit (Illumina, San Diego, CA) was used for FF samples and FFPE libraries were prepared with the a modified version of the TruSeq Nano kit.

Section S1.4. Whole genome sequencing and alignment
Sequencing was performed using Illumina HiSeq X (Genomics England cohort), HiSeq2500 (Oxford cohort) and a Novaseq 600 (PARTNER/PBCP cohort) to generate 150bp paired-end reads. Germline samples were sequenced to produce at least 85 Gb of sequences with sequencing quality of at least 30. For tumour samples at least 212.5 Gb were required. Alignments for the germline sample must cover at least 95% of genome at 15x or above with well mapped reads (mapping quality > 10) after discarding duplicates.

Section S1.5. Variant annotation

Genomics England
Illumina North Star pipeline (version 2.6.53.23) was used for primary small variant analysis. Tumour-normal subtraction and small variant calling were performed with Strelka (version 2.4.7).1 Structural variation and copy number variation were called using Manta (version 0.28.0) and Canvas (version 1.3.1), respectively. Reads were normalised via CellBase before small variant annotation using ENSEMBL (version 90/GRCh38), COSMIC (version v86/GRCh28), and ClinVar (October 2018 release). The GEL cancer pipeline allocates variants into domains based on the clinical utility of the gene. Domain one variants are defined as:

"variants in a virtual panel of potentially actionable genes in which small variants (SNVs and indels <50bp) have reported therapeutic, prognostic or clinical trial...associations, as defined by the GenomOncology Knowledge Management System."

Full details on the Genomics England's bioinformatic pipeline (including the list of Domain one variants) can be found int the documentation section of their website:
https://www.genomicsengland.co.uk/initiatives/100000-genomes-project/documentation

Oxford and PARTNER/PBCP Cohorts
Paired tumour-normal bam files were interrogated for somatic mutations using the following algorithms:

- CaVEMan for identification of somatic point mutations
  https://github.com/cancerit/CaVEMan
- Pindel for identification of small insertions and deletions
  http://cancerit.github.io/cgpPindel/

- BRASS for identification of somatic rearrangements https://github.com/cancerit/BRASS
- ASCAT for identification of somatic copy number changes https://github.com/cancerit/ascatNgs

Whole-genome sequencing (WGS) short reads were aligned to GRCh38/hg38 using BWA-MEM 0.7.17-r1188. Quality control and bioinformatic analysis of the WGS data was performed using CaVEMan23 (v1.13.15) for substitutions, Pindel24,25 (v3.2.0) for insertions/deletions, BRASS (v6.2.1) for rearrangements, and ASCAT (NGS) (v4.2.1) for copy number variations. Our full method of identifying somatically acquired copy-number alterations from whole genome sequencing data has been previously published.[1] Post-processing filters were applied to improve the specificity of mutation-calling. Specifically, for single nucleotide variant calls by CaVEMan23, we used CLPM = 0 and ASMD ≥ 140. To reduce false positive calls by Pindel24, we used QUAL ≥ 250 and REP < 10. For rearrangements called by BRASS, only those with assembly scores are considered.

Section S1.6. Quality Assessment

Sequence coverage and alignment metrics were calculated by Genomics England using inhouse tools or samtools (version 1.1). Tumour purity was estimated using Ccube.[2]

Section S1.7. Variant allele frequency and mutational burden

The distribution of VAF was assessed for all SNVs, substitutions, and indels. VAF was then normalised for tumour purity and assessed to investigate the effect of stromal contamination on VAF distribution. Indel mutational burden was calculated as the number of indels per sample per organ. Global mutational burden was assessed as somatic coding variants per Mb (reported by GEL).

Section S1.8. FFPEimpact formula

FFPEimpact score was calculated using the following formula:

$$FFPEimpact = \frac{\left(\frac{ID\ FFPE}{Total\ Indels} + \frac{(SBS57 + SBS\ FFPE)}{Total\ Substitutions}\right)}{2}$$

Where:

- *ID FFPE* is the exposure of the ID FFPE signature in the sample
- *SBS57* is the exposure of the SBS57 signature in the sample
- *SBS FFPE* is the exposure of the SBS FFPE signature in the sample
- *Total Indels* is the total number of indel mutations in the sample
- *Total substitutions* is the total number of substitution mutations in the sample

Section S1.9. Using FFPE indel artefact to salvage HRDetect

The HRDetect pipeline function from the signature.tools.lib (https://github.com/Nik-Zainal-Group/signature.tools.lib).[3] This function allows for flexible input specification to the HRDetect pipeline that computes the HRDetect score as published in Davies et al. 2017.[4] It requires an input data frame "data_matrix", which contains a sample in each row and one of six necessary features in each column. The six features can be computed by the pipeline if the necessary input files are provided. The six features are: 1) proportion of deletions at microhomology (del.mh.prop), 2) number of mutations of substitution signature 3 (SNV3), 3) number of mutations of rearrangement signature 3 (SV3), 4) number of mutations of rearrangemet signature 5 (SV5), 5) HRD LOH index (hrd), 6) number of mutations of substitution signature 8 (SNV8). For calculating the HRDetect score with FFPE samples, the proportion of deletions at microhomology were calculated following removal of artefactual patterns. The indel catalogues were generated for all the 578 samples. The exposures to indel signatures, including ID FFPE were then generated. The ID FFPE signature was then subtracted from the indel catalogues to create catalogues that have been “cleaned” of artefact. The proportion of deletions at microhomology (del.mh.prop) was then generated from the new catalogues and these values were input into the HRDetect pipeline.

Section S1.10. Calling of somatic copy number drivers

Somatic copy number drivers of two classes, amplification of an oncogene and homozygous deletion of a tumour suppressor gene were sought in a set of seven clinically important genes. An oncogene was considered amplified if the gene footprint overlaps with a region of total copy number above 5 for samples with a ploidy <2.5 and above 9 for samples with a ploidy >2.5. A tumour suppressor gene is considered homozygously deleted if the gene footprint overlaps with a segment of total copy number of 0 which is ≤1.5Mb long. The maximum length filter of deleted copy segments was required to avoid calling artefactual drivers. Homozygous deletion drivers were called only in samples of sufficient copy number call quality. 34% and 45% of FF and FFPE samples respectively were excluded on this basis due to over segmentation.

Tables

Supplementary Table 1: Overview of samples used for analysis – Genomics England Cohort

	Breast	CNS	Colorectal	Kidney	Lung	Ovary	Prostate	Uterus	Bladder	Total
**FF**	2509	504	2469	1355	1290	527	384	718	359	10115
**FF (PCR)**	283	76	113	95	114	60	84	43	31	899
**FFPE**	169	17	88	30	64	34	98	68	10	578

Supplementary Table 2: Overview of samples used for analysis – Oxford Cohort

	Breast	Colorectal	Kidney	Lung	Prostate	Uterus	Total
**FF**	10	12	13	5	4	7	51
**FFPE**	10	12	13	5	4	7	51

Supplementary Table 3: Overview of samples used for analysis – PARTNER/PBCP Cohort

	**Breast** (Triple Negative Breast Cancer)
**FF**	14
**FFPE**	14

Supplementary Table 4: Average sequencing coverage

	Genomics England		Oxford Cohort		PARTNER/PBCP Cohort	
	Tumour	Germline	Tumour	Germline	Tumour	Germline
**Minimum**	49.03	21.73	63.00	30.00	85.00	33
**1st Quartile**	93.02	30.69	79.25	34.50	96.75	37
**Median**	97.95	33.04	90.00	37.00	104.50	37
**Mean**	98.02	36.56	87.88	37.57	103.43	37
**3rd Quartile**	102.54	39.92	94.75	39.00	111.00	38
**Maximum**	175.96	158.47	123.00	61.00	118.00	39

Supplementary Table 5: Average coverage and alignment metrics (by sample preparation) – Genomics England Cohort

	Insert size	Chimeric percentage	Mapping rate	Coverage Heterogeneity	AT Bias	GC Bias	Tumour Purity
**FF**	477bp (460 to 494)	0.26% (0.21 to 0.34)	94.1% (93.0 to 95.3)	16.4 (15.8 to 17.0)	-2.8% (2.1 to 3.5)	- 1.6% (0.8 to 2.4)	48% (32 to 64)
**FF (PCR)**	475bp (459 to 491)	0.23% (0.19 to 0.28)	94.2% (93.2 to 95.2)	16.2 (15.5 to 16.9)	-3.9% (3.0 to 4.7)	-0.1% (-1.0 to 1.1)	48% (30 to 64)
**FFPE**	391bp (364 to 422)	0.51% (0.34 to 0.73)	93.4% (92.0 to 94.7)	26.3 (21.9 to 33.2)	-7.8% (5.9 to 10.0)	5.9% (-10.3 to -2.4)	45% (29 to 61)
**P-values**	FFvsFF(PCR) 0.0086 FFvsFFPE <2e-16	FFvsFF(PCR) 3.5e-08 FFvsFFPE <2e-16	FFvsFF(PCR) 0.54 FFvsFFPE <2e-16	FFvsFF(PCR) 0.29 FFvsFFPE <2e-16	FFvsFF(PCR) <2e-16 FFvsFFPE <2e-16	FFvsFF(PCR) <2e-16 FFvsFFPE <2e-16	FFvsFF(PCR) 0.43 FFvsFFPE 0.0018

The p-values in these tables were calculated using pairwise Wilcoxon rank-sum tests. A Benjamini-Hochberg (BH) correction was applied to adjust for multiple comparisons. All tests were two-sided.

Supplementary Table 6: Correlation of cancer cell content to VAF

		Spearman correlation	p-value
**EGFR variants**			
All samples	n=121	0.54	p=1.56e-10
FF	n=104	0.52	p=1.78e-08
FF(PCR)	n=9	0.53	p=0·14
FFPE	n=9	0.39	p=0.30
**KRAS G12C variant**			
All samples	n=144	0.66	p<2.2e-16
FF	n=127	0.66	p<2.2e-16
FF(PCR)	n=12	0.57	p=0·059
FFPE	n=5	0.9	p=0.083
**PIK3CA variants**			
All samples	n=974	0.53	p<2.2e-16
FF	n=818	0.52	p<2.2e-16
FF(PCR)	n=94	0.60	p=1.23e-10
FFPE	n=62	0.46	p=0·00016
**BRAF V600E variant**			
All samples	n=387	0.72	p<2.2e-16
FF	n=359	0.72	p<2.2e-16
FF(PCR)	n=17	0.76	p=0·00041
FFPE	n=11	0.81	p=0·0024

The correlation between variant allele frequency (VAF) and tumor purity was assessed using Spearman's rank correlation coefficient, which is a two-sided test.

Supplementary Table 7: Copy number driver event comparison between FF and FFPE (amplifications)

Cohort	ERBB2	FGFR1	GNAS	SOX2	CCND1
FF	7.7% (215/2792)	10.6% (298/2792)	6.4% (164/2582)	2.2% (31/1404)	6.9% (763/11,008)
FFPE	4.7% (8/169)	11.2% (19/169)	4.5% (4/88)	3.1% (2/64)	8.5% (49/578)
Organ	Breast	Breast	Colorectal	Lung	All

Supplementary Table 8: Copy number driver event comparison between FF and FFPE (homozygous deletions)

Cohort	PTEN	CDKN2A	MAP2K4
FF (n=7284)	2.1%	3.4%	1.1%
FFPE (n=316)	1.6%	3.8%	0.3%
Organ	All	All	All

Supplementary Table 9: Summary of SBS57 presence in FF samples

Cohort	Total sample number	Samples with SBS57	Median proportion of exposure	Interquartile range
FF	99052	0	-	-
FF (PCR)	572	57	25.05	10.36

Supplementary Table 10: Mutation burden comparison in Oxford and PARTNER/PBCP Cohorts

	p-value
**PARTNER/PBCP Cohort**	
Indels (FF vs FFPE)	p=1.2e-05
SNV (FF vs FFPE)	p=0·91
SV (FF vs FFPE)	p=0·11
**Oxford Cohort**	
Indels (FF vs FFPE)	p=2.0e-08
SNV (FF vs FFPE)	p=1.1e-05
SV (FF vs FFPE)	p=0·11

The p-values in these tables were calculated using pairwise Wilcoxon rank-sum tests. A Benjamini-Hochberg (BH) correction was applied to adjust for multiple comparisons. All tests were two-sided.

Supplementary Table 11: Driver Gene Mutations in Oxford Cohort

Gene	Number of gene mutations in FF samples	Number of gene mutations in FFPE samples
APC	18	8
ARID1A	8	8
ARID1B	4	3
ARID2	4	4
ATM	5	5
BCL11B	4	9
ERBB4	4	3
KRAS	8	7
LRP1B	5	6
MDM4	4	3
PBRM1	5	5
PIK3CA	13	12
PIK3R1	5	4
PTEN	11	12
PTPRD	4	2
SETD1B	4	4
SMAD3	4	3
STAT5B	5	5
TP53	21	19
VHL	6	6
LRP1B	0	1
PIK3R1	0	1

Supplementary Table 12: Matched Actionable Mutations in Oxford Cohort

Gene	Number of actionable mutations in FF samples	Number of actionable mutations in FFPE samples	Number of actionable mutations in FFPE samples matched to paired FF sample
AKT2	1	0	NA
APC	8	8	8
ARID1A	6	5	5
ATM	3	3	3
ATR	1	0	NA
BRAF	2	2	2
BRCA2	3	3	2
CCNE1	1	0	NA
CDK4	1	1	1
CDKN1B	2	2	2
CDKN2A	2	1	1
EGFR	1	0	NA
FBXW7	2	3	2
FGFR2	0	1	0
GATA3	1	1	1
IDH1	1	1	1
JAK1	0	1	0
KRAS	8	7	7
MET	1	0	NA
NF1	1	1	1
NF2	1	1	1
NOTCH1	0	1	0
NOTCH2	2	1	1
PBRM1	5	5	5
PIK3CA	11	9	9
PIK3R1	4	4	4
POLE	2	2	2
PTEN	7	8	7
RNF43	2	2	2
SETD2	3	2	2
STK11	1	9	1
TMPRSS2-ERG	2	2	2
TP53	8	7	7
VHL	6	6	6

Supplementary Table 13: Matched Domain 1 Mutations in PARTNER/PBCP Cohort

**Gene**	1	2	3	4	5	6	7	8	9	10	11	12	13	14
**AKT2**									FFPE					
**ATRX**			both											
**BRAF**			both						FFPE					
**BRCA1**	FFPE													
**EGFR**							FF							
**FGF21**			FF											
**FGFR2**													both	
**NRAS**	FF													
**PIK3R1**		FFPE												
**PTEN**					both									
**RAD54L**						both								
**RET**										FFPE		both		
**SMARCB1**												both		
**STK11**		both	both	both				both		both	both	both	both	both
**TP53**		both	both	both	both	both	both	both	both	both	both	both	both	both
**TSC2**	FF													

Supplementary Table 14: Overview comparison between FF and FFPE whole genome sequencing

	**FF**	**FFPE**
**Tumor**	Fresh frozen clinical sample	FFPE blocks, slides or scrolls<br><br>Permits DNA extraction from samples that have undergone routine diagnostics
**Germline surrogate**	Blood<br>Clinically healthy tissue	Blood only
**Cost**	$3000-5000 for WGS per patient	Additional considerations for FFPE:<br>1. Cost saving<br>- No requirement for cold-chain logistics in tissue transport and storage<br>2. Cost incurring<br>- FFPE specific DNA extraction kits<br>- Library preparation with enzymatic repair<br>- Higher sequencing depth may be required
**DNA input**	100-1000ng	250-600ng
**Sequencing metrics**	Average insert size: 477bp<br>Chimeric DNA: 0.26%<br>Mapping rate: 94.1%<br>Low duplication rate (0.08)	Average insert size: 391bp<br>Chimeric DNA: 0.51%<br>Mapping rate: 93.4%<br>Low duplication rate (0.12)
**Somatic variant call quality**	Gold standard<br>Background artefact (1%)<br>CN – 35% over segmented	SBS artefact – idiosyncratic<br>Indel artefact – Omnipresent<br>CN – 45% over segmented

Figures

Supplementary Figure 1: Tumour purity between different sample preparation methods

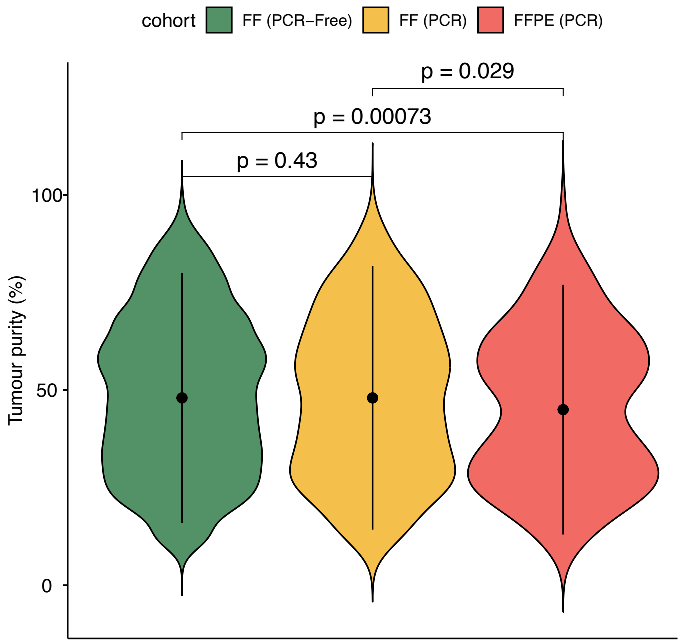

FF – n= 10,115 samples, Median= 48, IQR=32; FF (PCR) – n = 899 samples, median = 48, IQR = 33.75 ; FFPE – n = 578 samples, median = 45, IQR = 32. Wilcoxon rank-sum test used for statistical analysis.

Supplementary Figure 2: Proportional contribution of each organ type to total samples

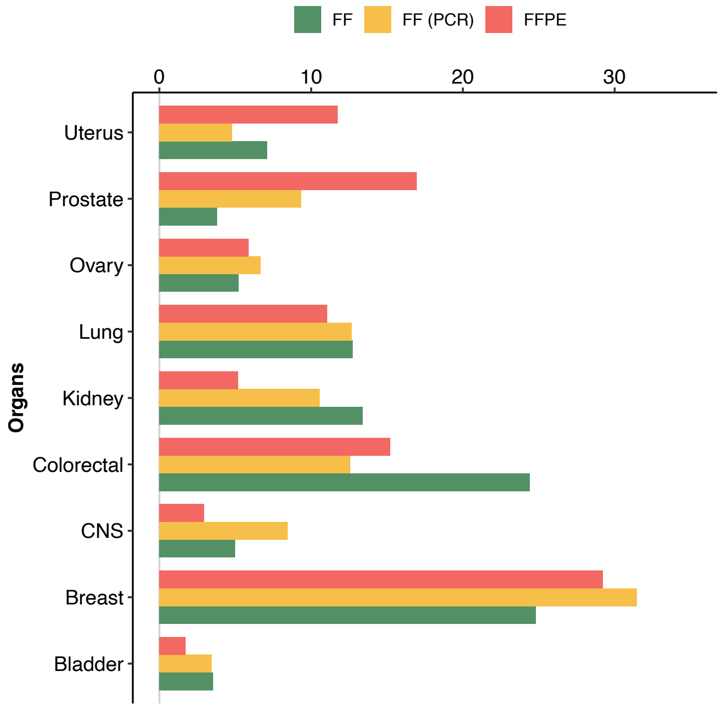

Kruskal-Wallis rank sum test - p-value = 0.46

Supplementary Figure 3: Somatic variants in potentially actionable genes

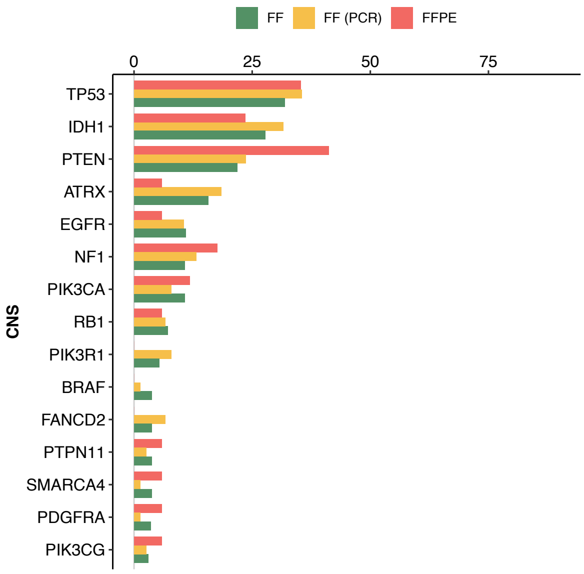

Kruskal-Wallis rank sum test - p-value = 0.95

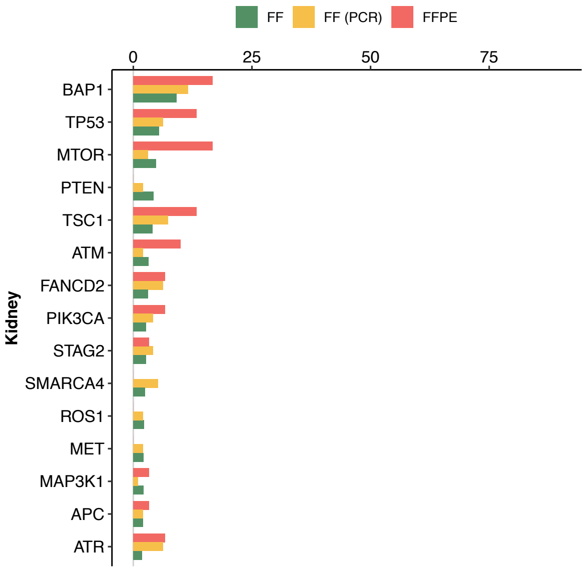

Kruskal-Wallis rank sum test - p-value = 0.35

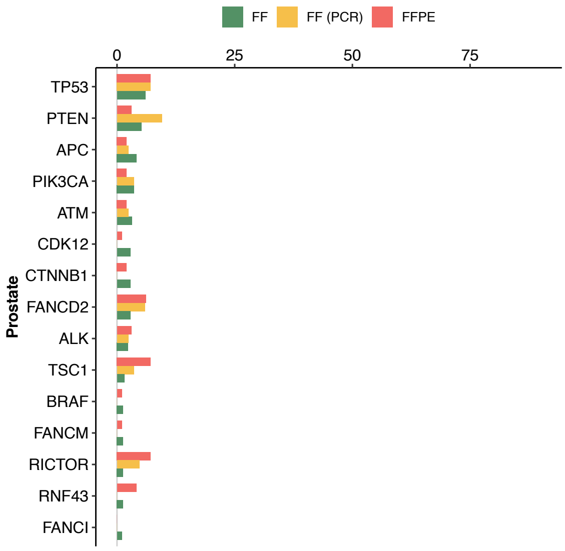

Kruskal-Wallis rank sum test - p-value = 0.36

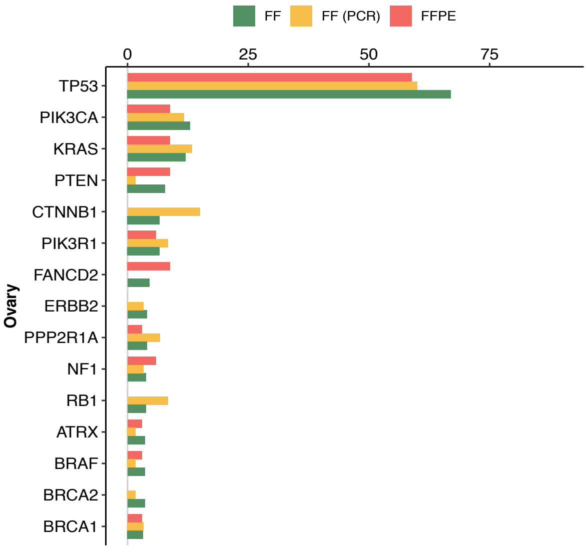

Kruskal-Wallis rank sum test - p-value = 0.80

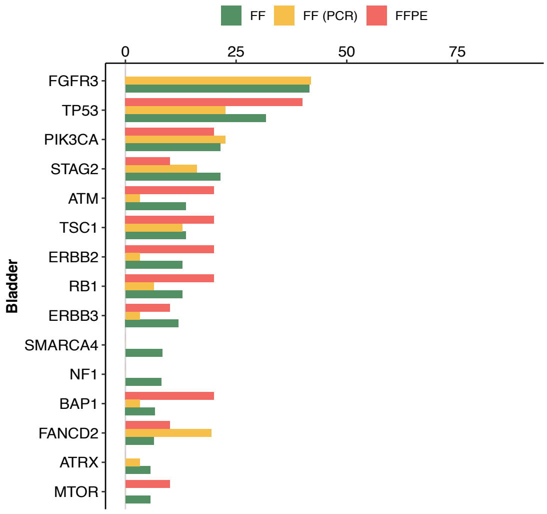

Kruskal-Wallis rank sum test - p-value = 0.38

Supplementary Figure 4: Relationship between artefactual signatures and FFPEimpact score

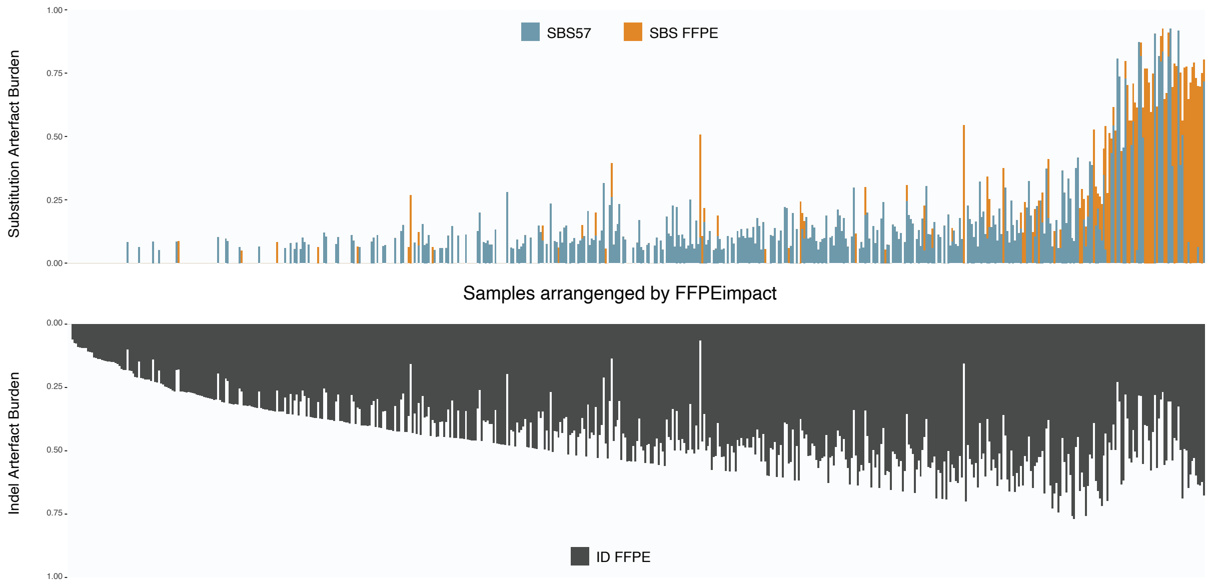

Supplementary Figure 5: Association between DNA extraction protocol and FFPEimpact score

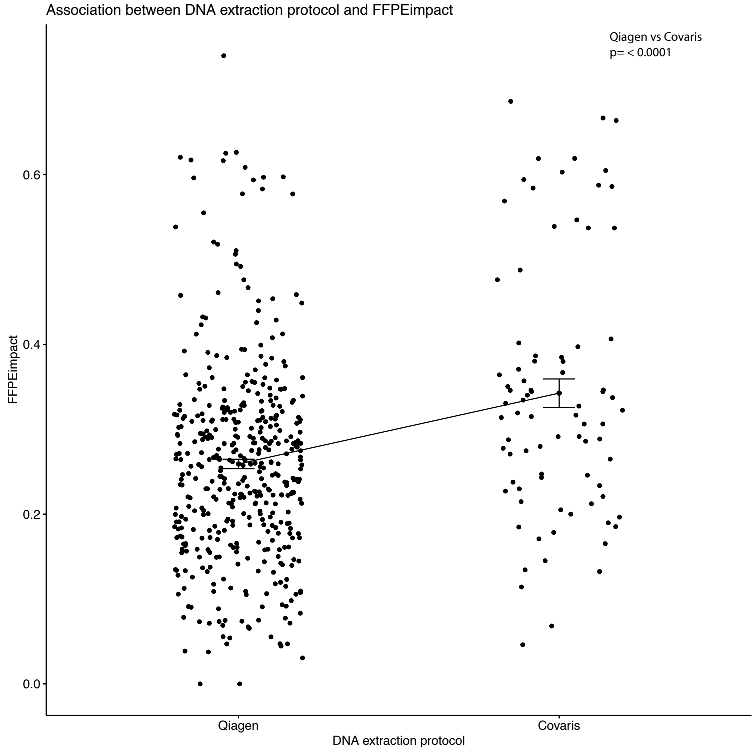

Association between DNA extraction protocol and FFPEimpact. Wilcoxon rank-sum test p = 1.4e-06

Supplementary Figure 6: Mutational burden analysis for Oxford cohort

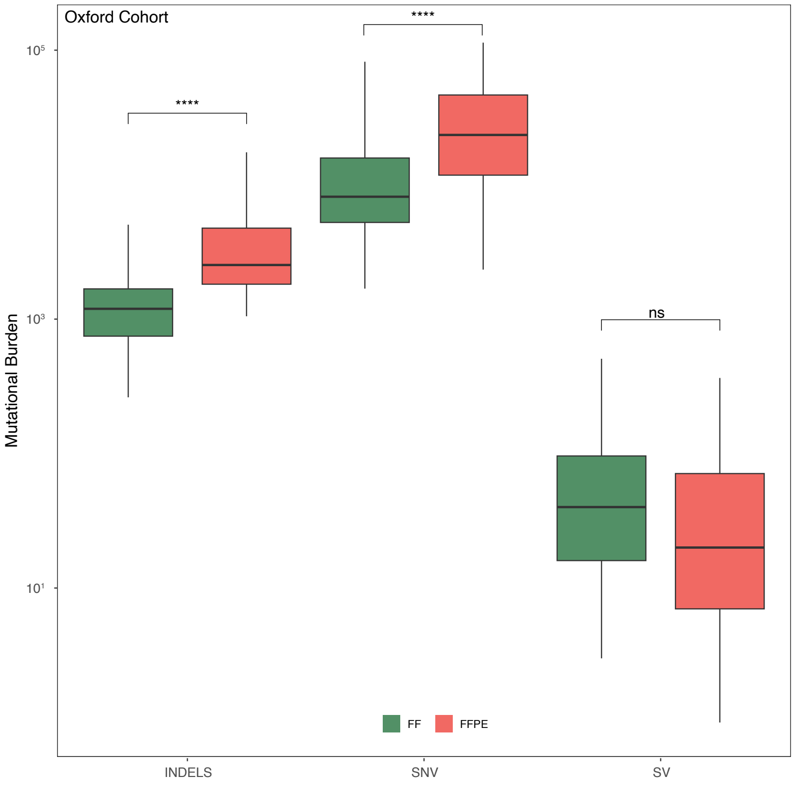

*Mutation burden of indels, SNVs and SVs in the Oxford cohort. Wilcoxon rank-sum test ns: p >0.05; *: p ≤ 0.05; **: p ≤ 0.01; ***: p ≤ 0.001; ****: p ≤ 0.0001. See Table S10 for exact p-values*

Supplementary Figure 7: Mutational burden analysis for PARTNER/PBCP cohort

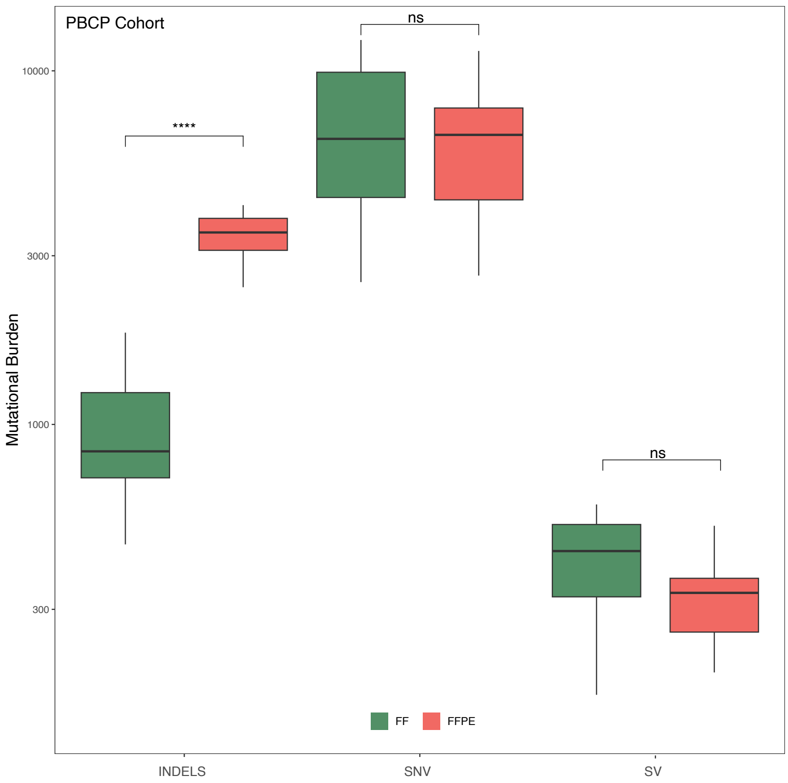

*Mutation burden of indels, SNVs and SVs in the PARTNER/PBCP cohort. Wilcoxon rank-sum test ns: p >0.05; *: p ≤ 0.05; **: p ≤ 0.01; ***: p ≤ 0.001; ****: p ≤ 0.0001. See Table S10 for exact p-values.*

Supplementary Figure 8: TruSeq FFPE research workflow

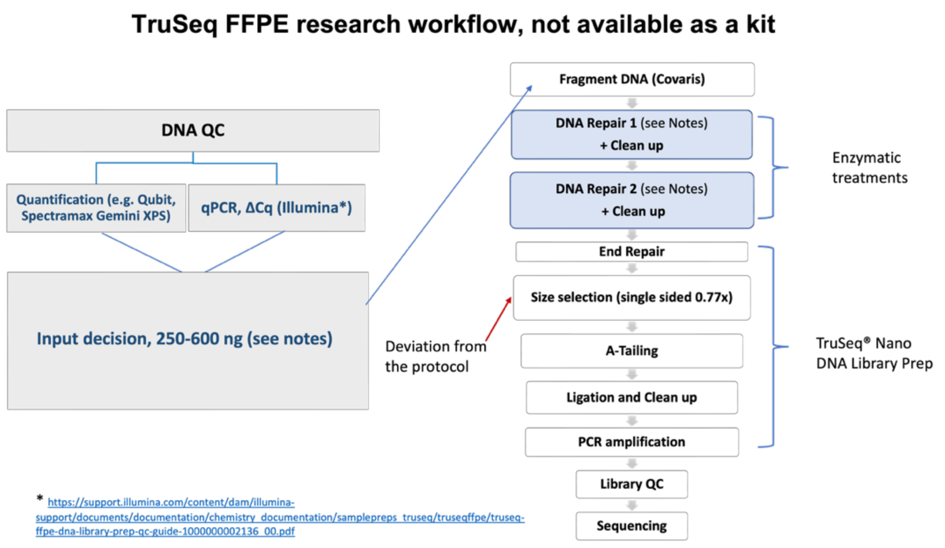

Notes:

Input decision
High ΔCq values are associated with poorer quality samples
Better quality samples have ΔCq values <1.0

In the GEL project, there was a cut-off of >2.5 for rejecting samples.
600 ng of DNA was used if available. For better quality samples 250 ng input was accepted if this was all that was available

DNA repair 1
Contains Uracil DNA Glycosylase and Endonuclease IV to remove deaminated cytosine residues and to hydrolyse the backbone at the abasic site

DNA repair 2
Contains RecJ to remove single stranded DNA

Full Collaborator Project Acknowledgements

Personalised Breast Cancer Program (PBCP)

Project team
(in alphabetical order, by surname)

Addenbrooke's: Jean Abraham (Chief Investigator); Shubha Anand; Steven Bell; Elizabeth Cromwell; James Drummond; Alex Fulton; Clare Hannon; Justine Kane; Ollie Kane; Jonathan Lay; Bin Liu; Rebecca Lucey; Meena Murthy; Karen Pinilla; Elena Provenzano; Philip Schouten; Victoria Theobald; Katrina (Jiaqi) Xian; Deborah Whitehorn; Joanna Worley

Illumina: Jennifer Becq; David Bentley; Terena James; Zoya Kingsbury; Martina Mijuskovic; Christine Rees; Mark Ross; Dunja Vucenovic;

Past members
(in alphabetical order, by surname)

Precision Breast Cancer Institute: Heather Biggs; Tracy Cook; Lynsey Drewett; Joanna Griffin; Emma Harrison; Linda Jones; Erica Law; Vanessa Moreira;
Cancer Molecular Diagnostics Laboratory: Mikel Valgañon
Medical Genetics: Patrick Tarpey; Howard Martin, Kim Brugger
Cancer Research UK Cambridge Institute: Chris Boursnell; Carlos Caldas; Kate Eason; Paul Edwards; Suet Feung-Chin; Raquel Manzano Garcia; Oscar Rueda;
Illumina: Sean Humphray; Tracey Saunby

Site Principal Investigators and team
(in alphabetical order, by surname)

Jennifer Abaddulay; Katrina Cooke; Mukesh Mukesh [Colchester]; Emma Hanbury; Nicky Levitt; Petra Troszt [Oxford]. Paul Ridley; Chris Rose; Liz Sherwin [Ipswich].

Acknowledgements
Primarily to the patients and the families and friends who supported them for participating in this study.

Also to: NIHR Cambridge BRC for their support for staff and infrastructure costs; The Cancer Research UK Cambridge Centre Cambridge for their support for staff and infrastructure costs; Cancer Molecular Diagnostics Laboratory for their support for sample collection; The clinical trials support staff at all participating sites.

Funders
Addenbrookes Charitable Trust Grant [9800]; The Mark Foundation for Cancer Research and Cancer Research UK [C9685/A25177]; Cancer Research UK [A27657]

PARTNER Trial

Project team
(in alphabetical order, by surname)

Current: Jean Abraham (Chief Investigator); Anita Chhabra; Elizabeth Cromwell; Alimu Dayimu; Erdem Demir; Nikolaos Demiris; Helena Earl; Steven Frost; Louise Grybowicz; Caron Harvey; Nicola Johnson; Karolina Lazarowicz; Rebecca Lucey; Camila Maida de pontes; Jessica Martin; Simon Morrison; Abirami Nandakopan; Karen Pinilla; Elena Provenzano; Nicola Robinson; Jeschito Serrano; Marc Tischkowitz; Anne-Laure Vallier; Nicky Watson; [Addenbrooke's Hospital].

Past: Angels Kateb Castellnou; Edmund Chui; Lynsey Drewett; Richard Hardy; Sian Loi; Emma McMurtry; Oluwatofunmi Okude; Wendi Qian; Anne Roberts; Stanly Thomas; Cherry May Sanchez; Melanie Weiss; Joanna Worley; Gemma Young.

Trial Management Group
Includes Project team members and the following site PIs (in alphabetical order, by surname):

Ellen Copson; Southampton General Hospital. Anne Armstrong; The Christie NHS Foundation Trust. Luke Hughes Davies; Addenbrookes Hospital. Karen McAdam; Peterborough City Hospital. Jacqueline Newby; Royal Free Hospital.

Site Principal Investigators
(in alphabetical order, by surname)

Shahzeena Aslam; Zaheer Abbas; Bedford Hospital. Sarah Ayers; Peterborough City Hospital. Annabel Borley; Velindre Cancer Centre. Gianfilippo Bertelli; Singleton Hospital. Jeremy Braybrooke; University Hospitals Bristol and Weston NHS Foundation Trust. Amit Chakrabarti; Poole Hospital. Steve Chan; Nottingham City Hospital. Mark Churn; Worcestershire Royal Hospital, Kidderminster Hospital and Treatment Centre. Maria Esther Una Cidon; Royal Bournemouth Hospital. David Mark Davies; Singleton Hospital. Daniel Glassman; Pinderfields Hospital. Tamas Hickish; Royal Bournemouth Hospital. Sreedevi Kumar; Pinderfields Hospital. Nicola Levitt; Churchill Hospital. Rasheid Mekki; Pinderfields Hospital. Margaret Moody; West Suffolk Hospital. Mukesh Mukesh; Colchester Hospital. Jay Naik; Pinderfields Hospital. Cheryl Palmer; Hinchingbrooke Hospital. Mojca Persic; Queen's Hospital Burton. Shazza Rahman; Auredake General Hospital. Sanjay Raj; Basingstoke and North Hampshire Hospital. Rebecca Roylance; University College Hospital. Liz Sherwin; Ipswich Hospital. Lucy Scott; University Hospital Crosshouse, University Hospital Ayr. Emma Staples; Queen's Hospital. Stephanie Sutherland; Mount Vernon Cancer Centre. Devashish Tripathi; Russells Hall Hospital. Ramachandran Venkitaraman; Ipswich Hospital.

Acknowledgements
Primarily to the patients and the families and friends who supported them for participating in this trial.

Also to: NIHR Cambridge BRC for their support for staff and infrastructure costs; The Cancer Research UK Cambridge Centre Cambridge for their support for staff and infrastructure costs; Cancer Molecular Diagnostics Laboratory for their support for sample collection; The Precision Breast Cancer Institute Team for their support for sample collection; The Cambridge Clinical Trials Centre – Cancer Theme for their support with core staff support; The clinical trials support staff at all participating sites.

Funders

Cancer Research UK [CRUKE/14/048]; AstraZeneca [1994-A093777]

References

1. Raine KM, Loo P, Wedge DC, et al. ascatNgs: Identifying Somatically Acquired Copy-Number Alterations from Whole-Genome Sequencing Data. Curr Protoc Bioinform 2016;56(1):15.9.1-15.9.17.

2. Yuan K, Macintyre G, Liu W, group P-11 working, Markowetz F. Ccube: A fast and robust method for estimating cancer cell fractions. Biorxiv 2018;484402.

3. Degasperi A, Zou X, Amarante TD, et al. Substitution mutational signatures in whole-genome–sequenced cancers in the UK population. Science 2022;376(6591).

4. Davies H, Glodzik D, Morganella S, et al. HRDetect is a predictor of BRCA1 and BRCA2 deficiency based on mutational signatures. Nat Med 2017;23(4):517–25.
