# Supplementary material for: Large-scale analysis of whole genome sequencing data from formalin-fixed paraffin-embedded cancer specimens demonstrates preservation of clinical utility
Source: Nat Commun. 2024 Sep 4;15:7731. doi: 10.1038/s41467-024-51577-2 (PMC11374794; doi:10.1038/s41467-024-51577-2)
Supplement: Supplementary file 3 — Description of Additional Supplementary Files [file 41467_2024_51577_MOESM3_ESM.docx]

**Description of Additional Supplementary Files**

**File Name: Supplementary Data 1**

Description: Sample Quality Metrics for Genomics England Cohort

**File Name: Supplementary Data 2**

Description: Single Base Substitution (SBS) and Indel Signatures Associate with FFPE artefact

**File Name: Supplementary Data 3**

Description: Single Base Substitution (SBS) Signature Exposures (Genomics England Cohort)

**File Name: Supplementary Data 4**

Description: Indel Signature Exposures (Genomics England Cohort)

**File Name: Supplementary Data 5**

Description: SBS57 exposure in FF(PCR) samples (Genomics England Cohort)

**File Name: Supplementary Data 6**

Description: HRDetect input matrix with and without correction for indel artefact (Genomics England Cohort)

**File Name: Supplementary Data 7**

Description: Average insert sizes and Copy number profiling (Oxford and PARTNER/PBCP Cohorts)

**File Name: Supplementary Data 8**

Description: FFPEimpact scores (Oxford and PARTNER/PBCP Cohorts)

**File Name: Supplementary Data 9**

Description: HRDetect and CHORD results for FF and FFPE samples (PARTNER/PBCP Cohort)

**File Name: Supplementary Data 10**

Description: Variants in Oxford and PARTNER/PBCP Cohorts
