# Supplementary material for: Large-scale analysis of whole genome sequencing data from formalin-fixed paraffin-embedded cancer specimens demonstrates preservation of clinical utility
Source: Nat Commun. 2024 Sep 4;15:7731. doi: 10.1038/s41467-024-51577-2 (PMC11374794; doi:10.1038/s41467-024-51577-2)
Supplement: Supplementary file 14 — Reporting Summary [file 41467_2024_51577_MOESM14_ESM.pdf]

nature portfolio

Corresponding author(s): Shadi Basyuni, Serena Nik-Zainal

Last updated by author(s): 21/11/23

Reporting Summary

Nature Portfolio wishes to improve the reproducibility of the work that we publish. This form provides structure for consistency and transparency in reporting. For further information on Nature Portfolio policies, see our Editorial Policies and the Editorial Policy Checklist.

Statistics

For all statistical analyses, confirm that the following items are present in the figure legend, table legend, main text, or Methods section.

n/a	Confirmed	
☐	☒	The exact sample size (*n*) for each experimental group/condition, given as a discrete number and unit of measurement
☐	☒	A statement on whether measurements were taken from distinct samples or whether the same sample was measured repeatedly
☐	☒	The statistical test(s) used AND whether they are one- or two-sided<br>*Only common tests should be described solely by name; describe more complex techniques in the Methods section.*
☐	☒	A description of all covariates tested
☒	☐	A description of any assumptions or corrections, such as tests of normality and adjustment for multiple comparisons
☐	☒	A full description of the statistical parameters including central tendency (e.g. means) or other basic estimates (e.g. regression coefficient) AND variation (e.g. standard deviation) or associated estimates of uncertainty (e.g. confidence intervals)
☐	☒	For null hypothesis testing, the test statistic (e.g. *F*, *t*, *r*) with confidence intervals, effect sizes, degrees of freedom and *P* value noted<br>*Give P values as exact values whenever suitable.*
☒	☐	For Bayesian analysis, information on the choice of priors and Markov chain Monte Carlo settings
☒	☐	For hierarchical and complex designs, identification of the appropriate level for tests and full reporting of outcomes
☐	☒	Estimates of effect sizes (e.g. Cohen's *d*, Pearson's *r*), indicating how they were calculated

Our web collection on statistics for biologists contains articles on many of the points above.

Software and code

Policy information about availability of computer code

Data collection	Somatic variants were identified in the 100,000 Genomes Project cohort according to the pipeline detailed by Genomics England, version 8 , without additional VAF-based filtering. For validation cohorts, FF and FFPE pairs were contrasted using the same bioinformatic pipeline: somatic single nucleotide variant (SNV) detection was performed with CaVEMan (Cancer Variants through Expectation Maximization: http://cancerit.github.io/CaVEMan/) and indel detection used Pindel (http://cancerit.github.io/cgpPindel/),20 again without additional VAF-based filtering.
Data analysis	Analysis was performed in R (version 4.0.3).<br>Mutational signature analysis was performed using the signature.tools.lib package (https://github.com/Nik-Zainal-Group/signature.tools.lib.git). FFPEimpact code for artefact has been made available online (https://github.com/Nik-Zainal-Group/FFPE_impact)

For manuscripts utilizing custom algorithms or software that are central to the research but not yet described in published literature, software must be made available to editors and reviewers. We strongly encourage code deposition in a community repository (e.g. GitHub). See the Nature Portfolio guidelines for submitting code & software for further information.

Data

Policy information about availability of data

All manuscripts must include a data availability statement. This statement should provide the following information, where applicable:

- Accession codes, unique identifiers, or web links for publicly available datasets
- A description of any restrictions on data availability
- For clinical datasets or third party data, please ensure that the statement adheres to our policy

Primary data from the 100,000 Genomes Project, which are held in a secure research environment, are available to registered users. See https://www.genomicsengland.co.uk for further information or contact M.A.B., Chief Scientific Officer at Genomics England (matt.brown@genomicsengland.co.uk).

Research involving human participants, their data, or biological material

Policy information about studies with human participants or human data. See also policy information about sex, gender (identity/presentation), and sexual orientation and race, ethnicity and racism.

Reporting on sex and gender	This study was an analysis of whole genome sequencing data from cancer specimens. Reporting on sex and gender was not needed to report on the required outcomes of the study
Reporting on race, ethnicity, or other socially relevant groupings	This study was an analysis of whole genome sequencing data from cancer specimens. Reporting on race,ethnicity or other socially relevant groupings was not needed to report on the required outcomes of the study
Population characteristics	Analysis was carried out on anonymised genomic data. Exact population characteristics does not impact anlaysis.
Recruitment	All patients were diagnosed with cancer in England's National Health Service and underwent a surgical procedure with curative intent. All provided written informed consent for WGS of tumour and a matched normal sample via the 100,000 Genomes Project and were recruited across all thirteen Genomic Medicine Centres in England. The recruitment cohort may have been biased to patients presenting to tertiary centres but this is unlikely to impact our results as our paper was a bioinformatic exercise comparing FFPE WGS with FF WGS
Ethics oversight	Samples analysed were collected as part of projects that were approved by relevant ethical committees and regulatory review bodies. Studies were the 100,000 Genomes Project (Genomics England) and the PARTNER/PBCP Programme (University of Cambridge)

Note that full information on the approval of the study protocol must also be provided in the manuscript.

Field-specific reporting

Please select the one below that is the best fit for your research. If you are not sure, read the appropriate sections before making your selection.

☒ Life sciences ☐ Behavioural & social sciences ☐ Ecological, evolutionary & environmental sciences

For a reference copy of the document with all sections, see nature.com/documents/nr-reporting-summary-flat.pdf

Life sciences study design

All studies must disclose on these points even when the disclosure is negative.

Sample size	No sample size calculation was used as this study was an analysis of existing whole genome sequencing data as part of Genomics England. 578 FFPE samples were analysed and compared to 11,014 FF samples. 3 datasets in total were used with two datasets acting as external validation. This was deemed sufficient as our analysis included samples from 3 different projects with many different sequencing protocols and platforms, thus mimicking the heterogeneity in clinical practice for cancer diagnostics.
Data exclusions	Nil
Replication	External validation cohorts were used to confirm reproducibility of data. 51 matched FFPE and FF samples from Oxford University Hospitals and 14 matched FFPE and FF samples from the PARTNER/PBCP programme in University of Cambridge were used. All replication attempts were successful.
Randomization	This study was an analysis of existing whole genome sequencing data as part of Genomics England - randomization was therefore not relevant
Blinding	This study was an analysis of existing whole genome sequencing data as part of Genomics England - blinding was therefore not relevant

Reporting for specific materials, systems and methods

We require information from authors about some types of materials, experimental systems and methods used in many studies. Here, indicate whether each material, system or method listed is relevant to your study. If you are not sure if a list item applies to your research, read the appropriate section before selecting a response.

Materials & experimental systems

n/a	Involved in the study
☒	☐ Antibodies
☒	☐ Eukaryotic cell lines
☒	☐ Palaeontology and archaeology
☒	☐ Animals and other organisms
☒	☐ Clinical data
☒	☐ Dual use research of concern
☒	☐ Plants

Methods

n/a	Involved in the study
☒	☐ ChIP-seq
☒	☐ Flow cytometry
☒	☐ MRI-based neuroimaging

Plants

Seed stocks	*Report on the source of all seed stocks or other plant material used. If applicable, state the seed stock centre and catalogue number. If plant specimens were collected from the field, describe the collection location, date and sampling procedures.*
Novel plant genotypes	*Describe the methods by which all novel plant genotypes were produced. This includes those generated by transgenic approaches, gene editing, chemical/radiation-based mutagenesis and hybridization. For transgenic lines, describe the transformation method, the number of independent lines analyzed and the generation upon which experiments were performed. For gene-edited lines, describe the editor used, the endogenous sequence targeted for editing, the targeting guide RNA sequence (if applicable) and how the editor was applied.*
Authentication	*Describe any authentication procedures for each seed stock used or novel genotype generated. Describe any experiments used to assess the effect of a mutation and, where applicable, how potential secondary effects (e.g. second site T-DNA insertions, mosiacism, off-target gene editing) were examined.*
